# Supplementary material for: Effectiveness of internet delivered cognitive behaviour therapy provided as routine care for people in the depressed phase of bipolar disorder treated with Lithium
Source: PLOS Digit Health. 2023 Feb 22;2(2):e0000194. doi: 10.1371/journal.pdig.0000194 (PMC9946241; doi:10.1371/journal.pdig.0000194)
Supplement: Attachment — Submitted filename: Response to reviewers PLOS digital iCBT for BD.docx [file pdig.0000194.s003.docx]

5 January, 2023

Danilo Pani, Ph.D.
Academic Editor
PLOS Digital Health

Dear Dr Pani

**PDIG-D-22-00173**: **Effectiveness of internet delivered cognitive behaviour therapy provided as routine care for people in the depressed phase of bipolar disorder**

Thank you for inviting us to submit a revised version of our paper. Our responses are in **bold** type.

Please review your reference list to ensure that it is complete and correct. If you have cited papers that have been retracted, please include the rationale for doing so in the manuscript text, or remove these references and replace them with relevant current references. Any changes to the reference list should be mentioned in the rebuttal letter that accompanies your revised manuscript. If you need to cite a retracted article, indicate the article's retracted status in the References list and also include a citation and full reference for the retraction notice.

**References checked and complete. Two additional references added in response to the suggestion of reviewer #1.**

2. Please send a completed 'Competing Interests' statement, including any COIs declared by your co-authors. If you have no competing interests to declare, please state "The authors have declared that no competing interests exist". Otherwise please declare all competing interests beginning with the statement "I have read the journal's policy and the authors of this manuscript have the following competing interests"

**The authors declare that no competing interests exist, now stated in the manuscript. (Prof Nielssen no longer holds ICTI shares, and doing so had no bearing on this study).**

Please amend your detailed Financial Disclosure statement. This is published with the article. It must therefore be completed in full sentences and contain the exact wording you wish to be published.

**Financial disclosure statement has also been amended.**

Please clarify all sources of funding (financial or material support) for your study. List the grants (with grant number) or organizations (with url) that supported your study, including funding received from your institution.

State the initials, alongside each funding source, of each author to receive each grant.
State what role the funders took in the study. If the funders had no role in your study, please state: “The funders had no role in study design, data collection and analysis, decision to publish, or preparation of the manuscript.”

If any authors received a salary from any of your funders, please state which authors and which funders.

If you did not receive any funding for this study, please simply state: “The authors received no specific funding for this work.”

**Sources of funding also clarified in the paper, which is that the authors received no specific funding for the study, including salary, grants or other support**

We have noticed that you have uploaded Supporting Information files, but you have not included a list of legends. Please add a full list of legends for your Supporting Information files after the references list.

**An index describing the supporting file is now included after the references.**

In the online submission form, you indicated that "All authors had access to all of the data. The datasets used and/or analysed during the current study available from the corresponding author on reasonable request". All PLOS journals now require all data underlying the findings described in their manuscript to be freely available to other researchers, either 1. In a public repository, 2. Within the manuscript itself, or 3. Uploaded as supplementary information.

**A spreadsheet with no information that might lead to the identification of an individual patient, but with sufficient information for other researchers to check the main findings, has been uploaded in the supplementary file.**

This policy applies to all data except where public deposition would breach compliance with the protocol approved by your research ethics board. If your data cannot be made publicly available for ethical or legal reasons (e.g., public availability would compromise patient privacy), please explain your reasons by return email and your exemption request will be escalated to the editor for approval. Your exemption request will be handled independently and will not hold up the peer review process, but will need to be resolved should your manuscript be accepted for publication. One of the Editorial team will then be in touch if there are any issues.

**Ethical approval requires that patient privacy be securely protected. Hence the full data set of patient demographic details and clinical response has not been uploaded. However, the supplemental file lists the psychotropic medications and symptom scores to allow the main finding to be checked.**

Additional Editor Comments (if provided):

The manuscript reports an interesting piece of research, however, the authors are kindly asked to improve the presentation by considering the attached reviews. In particular, please take into account the comments provided by the third reviewer, both in the manuscript and in rebuttal letter.

**The third reviewer’s comments do not set out any specific changes. Do they refer to the editorial requirements listed above?**

Reviewers' comments:

Reviewer #1: This is a well considered piece of research and a well written paper. Small things first - There are a few typos in the results section of the abstract and "effictiveness" later in the text.

**Thank you. Manuscript proof read again. Those typos corrected.**

Larger things - this is well done overall. My concern is that the conclusions that you state in the limitations section "with those limitations in mind.." (p17) are entirely fair and I think in order to accept this these should be the conclusions you quote in the Conclusions section of the abstract which are much more assertive and overly so in my opinion.

**Conclusions section of the Abstract amended accordingly.**

Selection bias and the impact of SES as a confounder are well known issues in the analysis of technology in healthcare and are probably at work here. There is huge class imbalance between the intervention and control group that is also typical of such analyses and could plausibly limit the generalizability of the findings here - might be worth citing this and discussing that these issues are not unique to the analysis of
this technology in the limitations section - [https://www.tandfonline.com/doi/full/10.1080/13696998.2021.1890416](https://protect-au.mimecast.com/s/s7OvCxngGkfox383I0bjFT?domain=tandfonline.com). With all that in mind I think it is reasonable to ask that the conclusions stated in the abstract are more conservative.

**Thank you for this interesting observation. Have added the recommended reference, and also a reference to our related service specifically targeting low SES patients, which achieved similarly good results for symptoms of depression. The message is that online treatments have the potential to overcome inequality of access to care.**

Reviewer #2: Dear Authors:
Thank you for the opportunity to review this interesting and important paper. Bipolar depression has a devastating impact on affected individuals and is notoriously difficult to treat. Further understanding of the effectiveness of internet delivered cognitive behavior therapy as part of routine care will fill gaps in current knowledge to help providers and patients manage this debilitating disease. This paper is well-written, clear, and – despite significant limitations – does advance the science of the management of bipolar depression. In the spirit of constructing as strong a paper as possible to convey this important information, I would like to offer some specific edits to the paper. I ask the authors to consider these suggestions not as overly focused on small details; rather in the spirit of creating the strongest, most succinct prose possible to highlight the strength of this paper.

Title: Please change the title to indicate that this study included only BDd patients who have been prescribed lithium. As the authors noted, this is a specific – but not sensitive – indicator of patients with BD. A large group of patients with BD have potentially been excluded from the study sample. It is important to clearly identify this in the title to assist with literature searches.

**Title amended to include reference to Lithium as recommended.**

Abstract: No suggested revisions

Author summary: The conclusion “The study shows that Lithium treatment is almost exclusively reserved for bipolar disorder in Australia” is a generalization not supported by the evidence provided in this study. Please either reword or remove this sentence.

**Sentence removed, to avoid controversy (although this information appears to be correct and is not available elsewhere).**

Introduction: The sentence which begins “However, once the diagnosis has been established, the depressed phase of BD is then assumed …” is awkward. Please consider rewriting the sentence to improve readability.

**Sentence amended, as recommended.**

Outcome measures: Please clarify why, if symptoms questionnaires were completed weekly, you chose to only use baseline and completion scores for analysis.

**A sentencing explaining the rationale has been added. Although the self report measures are sensitive to change, there is significant variability in the trajectory of individual patients.**

Table 1: Please ensure accuracy between the text and the information provided in Table 1. I noted several discrepancies. For example, the text states “Those with confirmed BD were older (43.9 years, SD 13.3 vs 39.8 years, SD 13.8),” however the table shows Confirmed BD 43.8 (13.3); the text states “Those with confirmed BD were … less likely to be employed (46.3% versus 61.2%),” however the table indicates Confirmed BD 49.2%. These are only 2 examples of discrepancies I noted in review of the concordance between text and table.

**Thank you for your attention to this detail. Now amended, and other quoted data points checked.**

Discussion: In the first sentence please include an indication that patients in the study were people who were prescribed lithium.

**Now included**

Please indicate that AD is an abbreviation for antidepressant.

**The full title for that abbreviation now included at first mention in the text.**

The statement “The results of this study suggest a greater emphasis should be placed on psychological treatments for BDd…” is presumptive. Consider something that better reflects the findings of this study within the context of the limited available evidence such as, “The results of this study suggest a prominent role for psychological interventions in the treatment of BDd …”

**Statement amended, as recommended.**

In the middle of page 17 there is an abbreviation that I believe is typed incorrectly as “DBb”

**Corrected.**

Thank you for the opportunity to review this excellent study. With the revisions noted here, I believe this paper would make a valuable contribution to the growing body of scientific literature on BDd treatment.

**Thank you**

Reviewer #3: Save for some observations which I believe can be sorted out, i think that the overall concept which informed the research is noteworthy and the result will help transform how we address the needs of theses group of patients. All other observations are as depicted in my critique. Thank you

Yours sincerely

Olav Nielssen
