# Supplementary material for: Effectiveness of internet delivered cognitive behaviour therapy provided as routine care for people in the depressed phase of bipolar disorder treated with Lithium
Source: PLOS Digit Health. 2023 Feb 22;2(2):e0000194. doi: 10.1371/journal.pdig.0000194 (PMC9946241; doi:10.1371/journal.pdig.0000194)
Supplement: Attachment — Submitted filename: Manuscript Critique (3).docx [file pdig.0000194.s004.docx]

Manuscript Critique

MANUSCRIPT TYPE: PLoS Digital Health

Manuscript Number: PDIG-D-22-00173

**Full Title**: Effectiveness of internet delivered cognitive behaviour therapy provided as routine care for people in the depressed phase of bipolar disorder

Suggested: Effectiveness of routine internet delivered cognitive behaviour therapy in depressed bipolar disorder or Effectiveness of internet delivered cognitive behaviour therapy provided as routine care for people with depressed bipolar disorder

**Abstract :**

: I expect authors to give a brief summary of what CBT is.

The necessity and successes or otherwise of CBT

I expect them to also briefly give the outcome of CBT in Depressed Bipolar

The title suggests that some research exist. So, why is this different?

This would justify why CBT or iCBT is necessary.

**METHODS**

It would seem that only those using Lithium were selected even though the researchers acknowledged that others used other standard forms of treatment. Why were those ones excluded? How can the result be generalized if these group of persons are not part of the research?

Completion rate was an outcome? Of the iCBT? How is that related to wellness or resolution of the symptoms? or re the inferring that every single person who completed the programme got well?

Your research topic was solely about depressed phase of BAD, why measuring Anxiety? Or are you saying that depression must be accompanied by anxiety in all cases?

**RESULTS**

In the same vein, what happens if there are remnant of anxiety symptoms and no depressive symptoms? Would the patient be said to be well? At what cut-off would one say the intervention is successful?

How did the researchers having acknowledged the debilitating effects of medication exclude Lithium as a confounder?

How did they exclude anxiety as a confounder?

**CONCLUSION**

This mentions anxiety again, as though it is synonymous with depression. Was the intervention for both conditions? This is because there is a clear cut subgroup of patients with depressed BAD with anxiety.

**OVERALL**

There appears to be some gaps in the overall aim and objectives and a fusion of the expectation. It is not clearly demonstrated what the end point is and how to measure them.

**COMPETING INTEREST**

**3 out of 6 researchers have both direct and indirect interest in the intervention. It’s a difficult sell.**
